# “Who I am”: understanding the self-identity process of autism in adults in the UK

**DOI:** 10.1080/17482631.2026.2682104

**Published:** 2026-06-02

**Authors:** Gayle L. Overton, Ferran Marsà-Sambola, Rachael Martin, Penny Cavenagh

**Affiliations:** a School of Social Sciences and Humanities, Psychology, University of Suffolk, Ipswich, Suffolk, UK; b School of Psychology and Education, Open University of Catalonia, Barcelona, Catalunya, Spain; c Hertfordshire Partnership NHS Foundation Trust, Mid and West Essex ASD Diagnostic Service, Hatfield, Hertfordshire

**Keywords:** Autistic identity, self-identification, diagnosis, ASIS, neurodiversity, focus groups, autism spectrum

## Abstract

**Purpose:**

This study aimed to: (1) explore factors shaping the self-identification process among adults who identify as autistic; (2) examine how self-identification functions in relation to formal diagnosis, including whether it serves as a sufficient endpoint or a transitional stage; and (3) contribute to the UK adaptation of the Autism Spectrum Identity Scale (ASIS) as a complementary identity-focused measure within adult assessment contexts.

**Methods:**

Twelve UK-based adults participated in one of two online focus groups: six self-identified autistic adults without formal diagnosis and six adults who were both self-identified and formally diagnosed. Data were analysed using reflexive thematic analysis. Participants completed a cognitive review of the ASIS to inform adaptation.

**Results:**

Self-identification emerged as a dynamic, context-dependent process shaped by lifelong experiences of difference, masking, misrecognition, and structural barriers to diagnosis. For some participants, self-identification constituted a sufficient identity position; for others, it functioned as a catalyst for pursuing formal diagnosis to access validation or support. Identity development and co-occurring mental health conditions were described as interacting in bidirectional ways.

**Conclusions:**

Self-identification does not replace formal diagnosis but interacts with it in relational and context-sensitive ways. Incorporating identity-oriented dialogue into assessment processes may enhance person-centred practice.

## Introduction

Autism Spectrum Disorder (ASD), as defined by the DSM-5TR (American Psychiatric Association, 2022), is a neurodevelopmental condition characterised by persistent difficulties in social communication and interaction, alongside restricted and repetitive patterns of behaviour, interests, or activities. This definition is grounded in the medical model, which primarily conceptualises autism in terms of deficits (Chapman, 2021). However, there has been a growing shift toward a neurodiversity-affirming approach, which views autism as a natural and valuable variation in human neurology (Pellicano & Den Houting, 2022).

There has been growing interest in the lived experiences of autistic adults, especially those who remain undiagnosed or self-identify as autistic. Many such individuals face significant challenges in obtaining a formal assessment or diagnosis, including long waitlists, geographic limitations in service provision, and clinician bias.

In this study we refer to “accessing diagnostic services” rather than simply “being diagnosed”, to pinpoint the systemic impediments to formal assessment.

In this context, we acknowledge that diagnostic criteria retain an important role in access to services and recognition of needs, while also recognising their limitations in capturing the full spectrum of autistic identity. In line with Overton et al. (2024), we do not position self-perception as a replacement for formal diagnosis, but rather as a complementary route to self-understanding, particularly when diagnostic routes are blocked or delayed.

Such barriers are especially pronounced for adults assigned female at birth (AFAB), LGBTQ+ individuals, and people from culturally or socioeconomically marginalised backgrounds (Crane et al., 2018; Mezzatesta Gava et al., 2025; Muggleton et al., 2019). In adult autism assessment specifically, additional challenges arise, for example, retrospective recall of early developmental history is often required, many standard instruments were designed for children and later adapted for adults, and overlapping mental health conditions may obscure autistic traits. Masking, understood as the suppression or camouflaging of autistic traits in social settings (Klein et al., 2024), further complicates recognition and assessment. The heterogeneity of presentation, including overlap with anxiety, depression or other diagnoses, and differences in female or later recognised phenotypes of autism, also contributes to under identification.

The combination of co-occurring mental health conditions, masking, diagnostic limitations and under recognised adult presentations has led to the notion of a “lost generation” of autistic adults whose identities went unrecognised earlier in life (Lai & Baron-Cohen, 2015; Scattoni et al., 2021). Consequently, many adults pursue alternative routes to understanding themselves, often turning to self-identification as a meaningful, though unofficial, process of recognition.

In this study, we define self-identification in two analytically distinct but related senses. First, *epistemic self-recognition* refers to the process by which individuals come to interpret their experiences through an autistic framework. Second, *identity integration* refers to the incorporation of autism as a valued and coherent aspect of self-concept (Overton et al., 2024). These processes are shaped both by personal meaning-making and by structural conditions, such as access to assessment, clinician gatekeeping, sociocultural narratives, and service availability. Self-identification may precede, accompany, follow, or in some cases function independently of formal diagnostic processes. Rather than assuming that self-identification is inherently transitional or inherently sufficient, we approach it as context-dependent and relational.

These two dimensions are not always sequential or separable, and the study does not pursue them as a formal twofold analytic structure. Rather, both emerged across participants’ accounts as intertwined aspects of a broader meaning-making process: some individuals recognised themselves as autistic long before integrating that understanding into a stable sense of self, whilst others described identity integration as preceding, or occurring independently of, explicit epistemic clarity. The distinction is retained insofar as it illuminates qualitative differences in how participants described their self-identification trajectories, not as a framework that requires two discrete corresponding answers.

According to Moore (2016), self-identification is often a gradual and multifaceted process. It typically unfolds through several stages, beginning with an initial suspicion that one might belong to a particular group or identity category. This is followed by a period of information seeking, during which individuals gather knowledge and compare their experiences with others. Tentative self-identification often emerges next, as people begin to test and explore this possible identity. The process then extends to disclosure to others, where individuals share their self-understanding in interpersonal or community contexts. Finally, many reach a phase of identity integration or pride, in which the new identity is internalised as a positive and coherent aspect of the self.

Although the emerging literature acknowledges this phenomenon, the concept of autistic self-identification remains under-researched. Some studies show that most self-identified autistic adults tend to be female and in midlife (Lewis, 2017; Overton et al., 2024). Additionally, research on female and gender diverse phenotypes has broadened recognition of diverse presentations and contributed to increased self-advocacy among adults (Pantazakos & Vanaken, 2023).

However, few studies have explored how self-identification relates to formal diagnosis, or how these processes interact over time. A central aim of this study is therefore to examine how adults understand the relationship between self-identification and formal diagnosis, including whether self-identification may function as a stable identity position for some individuals or as a step toward pursuing clinical assessment for others.

The preceding sections have outlined the structural and personal dimensions of self-identification and its relationship to formal diagnosis. A complementary strand of enquiry concerns how existing psychological instruments engage with, and may shape, autistic identity. Recent scholarship has emphasised the importance of co-produced and neurodivergent-affirming tools in autism research and assessment (Overton et al., 2024). Many widely used assessment measures for adults were originally developed for children and later adapted and may not fully reflect how adults describe their internal experiences, identity processes, and contextual challenges. Instruments commonly used in clinical pathways are designed to identify behavioural indicators consistent with diagnostic criteria, but they may not capture how adults understand themselves or integrate autism into their broader sense of identity. This limitation is directly relevant to the processes described above: if the language of measurement fails to resonate with autistic adults’ self-understanding, it risks reinforcing the very misrecognition that delays or complicates self-identification.

The Autism Spectrum Identity Scale (ASIS) was developed by McDonald (2017, 2020) as a self-report instrument to measure variation in autism identity. It is not a diagnostic tool and does not determine whether an individual meets criteria for autism. Rather, it assesses identity-related dimensions across four domains: positive difference, context dependency, spectrum abilities, and changeability. Higher scores in positive difference, changeability, and spectrum abilities reflect a more positive autism identity. The ASIS has demonstrated good psychometric properties in United States samples (McDonald, 2017, 2020).

With permission from the original author, the present study contributes to the qualitative development of a UK-adapted version of the ASIS. Our intention is not to replace existing NICE-recommended diagnostic instruments, nor to position the ASIS (McDonald, 2017) as a stand-alone assessment tool. Instead, we explore how an identity-focused measure may complement clinical assessment by prompting reflection on lived experience, facilitating dialogue, and broadening understanding of how adults conceptualise autism beyond behavioural criteria. A separate publication will report the psychometric evaluation of the UK version following this qualitative adaptation phase.

In the UK, the ASIS (McDonald, 2017) has not been widely evaluated among self-identified or UK-based adults and may contain language or assumptions that do not fully align with neurodiversity-affirming perspectives or contemporary adult discourse.

The inclusion of both self-identified and formally diagnosed participants allows us to examine whether identity processes and evaluations of the ASIS (McDonald, 2017) differ according to diagnostic status, and to explore how structural access to diagnosis may shape identity construction and engagement with identity measures. The two groups were not conceived hierarchically but comparatively, so as to examine relational dynamics between self-identification and diagnosis.

Thus, this study addresses three interrelated aims:

(1) Explore the main factors involved in the self-identification process of autism in adults.
(2) Understand how self-identification functions in relation to formal diagnosis, including whether it may serve as a sufficient endpoint for some adults or as a transitional stage for others.
(3) Evaluate, through co-produced qualitative enquiry, the development of a UK-adapted version of the ASIS (McDonald, 2017) as a complementary identity-focused tool within adult autism assessment contexts.

## Method

### Design and approach

This study adopted a qualitative design using focus groups and reflexive thematic analysis, supported by a collaborative, neurodiversity-affirming framework.

The study was informed by an interpretivist epistemological stance, recognising autistic identity as socially constructed, relational, and shaped by structural conditions. In line with the conceptual framing outlined in the Introduction, we approached self-identification as a dynamic process that may unfold differently depending on access to formal diagnosis and personal meaning-making.

The research was co-produced with two autistic collaborators who contributed to question development, thematic analysis, and interpretation. Their insights shaped the research at each stage and ensured that the study reflected lived experience perspectives.

The inclusion of two groups defined by diagnostic status was analytically intentional. This design enabled exploration of whether self-identification unfolds differently depending on structural access to diagnosis, and whether formal diagnostic confirmation modifies identity integration, validation, or engagement with identity measures. The two groups were not conceived hierarchically but comparatively, allowing examination of relational dynamics between self-identification and diagnosis.

### Researcher positionality

All authors of this study have clinical, social, educational, and lived experience related to autistic adults. Autistic collaborators were actively involved throughout the project. They co-generated the research questions, co-designed the focus group materials and ASIS review procedures, reviewed and piloted the interview and focus group guides to ensure plain-language clarity, participated in coding and thematic interpretation meetings, and contributed to draughting and editing the analysis and discussion sections. Their involvement was continuous and documented in project records. Their lived experience, accumulated over four decades, provided critical insight, particularly in analysing how identity is formed and negotiated over time.

We acknowledge that our neurotype, disciplinary backgrounds, and social contexts inevitably shape the interpretive process. This positionality introduces perspectives while also enriching the analytic lens through which findings are interpreted.

### Participants

Twelve UK-based adults participated in the study: nine female, two male, and one agender individual, aged between 20 and 58 years, with a mean age of 39. All participants reported co-occurring conditions, including anxiety, ADHD, PTSD, eating disorders, depression, sleep-related challenges, and dyslexia. A summary of sociodemographics is shown in Table I.

**Table I.** Sociodemographic characteristics of participants.

Name (Pseudonym)	Gender	Age	Diagnosis status	Occupation	Co-occurring conditions	Locality (UK region)
Alex	Agender	25	Diagnosed	Assembly Line Worker	Dyslexia	Northwest England
Phil	Male	20	Diagnosed	Software Developer	Anxiety	Southeast England
Sophia	Female	30	Diagnosed	Data Analyst	Depression	London
Grace	Female	36	Diagnosed	Accountant	ADHD	East Midlands
Olivia	Female	40	Diagnosed	Librarian	ADHD	Scotland
Hannah	Female	45	Diagnosed	Technical Writer	Sleep Problems	Southwest England
Lily	Female	50	Self-Identified	Scientist	PTSD	Northeast England
Abigail	Female	55	Self-Identified	Graphic Designer	Eating Disorder	Wales
Charlotte	Female	45	Self-Identified	Lab Technician	Substance Use Disorder	East of England
Zoe	Female	38	Self-Identified	Veterinary Technician	Anxiety	Southwest England
Jamie	Female	50	Self-Identified	Quality Inspector	Depression	Midlands
Oliver	Male	45	Self-Identified	Tech Support Specialist	ADHD	Norther Ireland

Participants were assigned to one of two online focus groups based on diagnostic status:

Group One: Six adults who self-identified as autistic but had no formal diagnosis.

Group Two: Six adults who were both self-identified and formally diagnosed as autistic.

The separation into two groups was intended to explore whether structural access to diagnosis influenced identity development and perceptions of assessment tools. This grouping allowed examination of similarities and differences in identity construction processes while maintaining analytical sensitivity to diagnostic status as a contextual factor rather than a marker of legitimacy.

Participants were recruited via an online advert circulated through autism-focused community pages, UK autism organisations, and social media platforms. The advert included accessibility information and signposted support resources.

Eligibility criteria were age 18 years or older, residence in the UK, self-identification or formal diagnosis of autism as per group allocation, and capacity to consent. Exclusion criteria included current severe crisis or other acute mental health risk identified during screening.

### Ethics

Ethics approval for this study was obtained through the Research Ethics Committee at the University of Suffolk. Informed written consent was obtained from all participants prior to participation.

### Procedure

Participants completed screening and informed consent prior to final enrolment. Consent materials were emailed in advance and verbal consent was reconfirmed at the start of each session.

Each focus group lasted approximately 90 minutes and was conducted online via a secure videoconferencing platform. Both groups were facilitated by the lead researcher, an experienced qualitative researcher with clinical expertise in autism, who moderated all discussions. The facilitator used the co-produced question guide to structure discussion, employed open-ended follow-up probes to deepen responses (e.g., “Could you say more about that?”, “Has anyone had a similar or different experience?”), and drew on active listening and reflective techniques to encourage equitable participation. Discussion unfolded iteratively rather than rigidly following the question sequence; participants were encouraged to respond to one another, and the facilitator introduced questions at natural junctures to maintain conversational flow. Participants received advance materials including a session outline, accessible instructions for using the platform, and discussion topics. This preparation aimed to reduce anxiety and facilitate reflective contributions. The sample size of twelve participants across two groups was considered appropriate for a qualitative, interpretivist study of this scope, consistent with established guidance on focus group research (Krueger & Casey, 2015), which prioritises analytic depth and purposive composition over numerical breadth.

Accessibility adaptations included chat-based responses, the option to respond privately to the facilitator, pre-circulated materials, scheduled breaks, and the possibility of a one-to-one follow-up if preferred. Private chat responses were included in the data corpus and treated in the same way as spoken contributions, preserving anonymity and ensuring that less verbally communicative participants were equally represented.

### Focus group questions

Six open-ended questions were co-produced for discussion:

1. What preceded the self-identification process of autism?
2. What were the main routes linked with this process?
3. What were the main confirming factors?
4. What feelings were associated with self-identification?
5. How long did the process take?
6. What triggered or prevented a formal assessment?

These questions were informed by existing models of self-identification and shaped through input from lived experience collaborators.

### ASIS (McDonald, 2017) instrument review

Following each focus group, participants engaged in a structured item-by-item review of the Autism Spectrum Identity Scale. The ASIS (McDonald, 2017) was originally developed by McDonald as a self-report instrument measuring variation in autism identity across four domains: positive difference, context dependency, spectrum abilities, and changeability.

The ASIS (McDonald, 2017) is not a diagnostic instrument and does not determine whether an individual meets criteria for autism. Instead, it measures dimensions of autism-related identity. With permission from the original author, this study contributes to the qualitative development of a UK-adapted version of the ASIS (McDonald, 2017). This phase focused on cultural and linguistic adaptation through co-produced qualitative feedback. A separate study will report the psychometric evaluation of the UK version prior to implementation in research or clinical contexts.

Participants were invited to comment on clarity, relevance, tone, inclusivity, and conceptual framing. Participants were informed that the ASIS (McDonald, 2017) is not intended to replace NICE-recommended diagnostic tools but may serve as a complementary identity-focused instrument capable of prompting reflection and dialogue within adult assessment contexts.

Feedback was recorded, transcribed, and analysed thematically.

### Data analysis

All transcripts were analysed using Braun and Clarke's (2006) six-phase framework for reflexive thematic analysis. A combined semantic and latent coding approach was applied to capture explicit meanings and underlying patterns of sense-making.

The analytic team included two primary coders and two autistic collaborators who participated in analytic meetings to review codes, refine themes, and interrogate interpretations. Disagreements were addressed through discussion and consensus, with the reflexive journals maintained throughout the process serving as a record of interpretive decisions. Regular meetings addressed how researcher positionality and assumptions might shape interpretation.

Analytic attention was paid to how participants described the relationship between self-identification and formal diagnosis, and how co-occurring conditions intersected with identity construction. These dimensions were examined both within and across the two diagnostic-status groups.

ASIS (McDonald, 2017) feedback was analysed separately but in parallel, using thematic coding informed by cognitive interviewing principles. To maintain methodological clarity, the focus group thematic analysis drew on the verbatim transcripts of the open-ended discussion (questions 1–6), while the ASIS review drew on participants' item-by-item commentary recorded during the structured cognitive review phase. Although both datasets were generated within the same sessions, they were coded separately to preserve the analytic distinction between autobiographical narrative and instrument evaluation.

## Results

### Thematic analysis

Six overarching themes emerged from participants' accounts, tracing a progressive narrative from early experiences of difference to the development of autistic identity and pride.

### Theme 1: experiencing difference in a neurotypical world

This theme explores participants' enduring sense of being “different” or “out of sync” with societal expectations, a feeling that often predated any awareness of autism.

*“I always felt like I missed a memo that everyone else got about how to be a person.”* (Zoe, self-identified)

*“Even as a kid, I thought something was 'off,' but no one else saw it.”* (Phil, diagnosed)

*“It was like everyone had the rulebook but me.”* (Charlotte, self-identified)

*“I've always felt like an alien trying to learn the rules of a planet I wasn't from.”* (Alex, diagnosed)

Self-identified participants often arrived at recognition through online communities or autism-related literature, whereas diagnosed individuals described parallel realisations that typically followed years of misdiagnosis.

*“Therapy after therapy, and no one ever asked if it could be autism.”* (Sophia, diagnosed)

*“TikTok was the lightbulb moment. Suddenly everything made sense.”* (Lily, self-identified)

*“I didn't discover autism. I discovered myself through it.”* (Jamie, self-identified)

### Theme 2: diagnosis and the construction of identity

Diagnosis (formal or self-conferred) emerged as a pivotal identity milestone. Self-identified participants emphasised autonomy and self-knowledge, while those with formal diagnoses described the process as validating yet emotionally complex.

*“I don't need a certificate to know who I am.”* (Charlotte, self-identified)

*“Diagnosis gave me a language for what I'd always felt but couldn't explain.”* (Grace, diagnosed)

*“It validated everything I'd been gaslit into doubting.”* (Phil, diagnosed)

Some self-identified individuals expressed distrust toward clinical assessment pathways due to previous misdiagnoses or experiences of professional gatekeeping:

*“My GP literally said, 'You don't look autistic.' That killed it for me.”* (Lily, self-identified)

*“They told me I was too social to be autistic. Whatever that means.”* (Jamie, self-identified)

*“I'd already been misdiagnosed three times. I stopped trusting the system.”* (Oliver, self-identified)

All self-identified participants reported prior misdiagnoses (e.g., depression, anxiety, ADHD); we report these frequencies in Table I and discuss below how prior misdiagnoses contributed to delayed recognition and self-directed identity work.

Diagnostic inaccuracies often delayed or complicated identity construction. Participants described feeling invalidated or mislabeled, which reinforced masking and postponed access to identity-affirming communities. For example, one participant noted: “Therapy after therapy, and no one ever asked if it could be autism.” (Sophia, diagnosed), illustrating how misdiagnosis can obscure identity formation.

Across both groups, self-identification did not function uniformly in relation to formal diagnosis. Among self-identified participants, some described self-identification as a sufficient and stable identity position, particularly when structural barriers or prior negative clinical experiences reduced the perceived value of pursuing formal assessment. Others described it as a provisional stage that motivated them to seek diagnosis for validation, workplace accommodations, or access to services.

Among formally diagnosed participants, self-identification typically preceded diagnosis and facilitated the decision to pursue assessment. Diagnosis was described as providing institutional recognition and access to support, but not as generating identity itself. Identity development was already underway prior to diagnostic confirmation.

These accounts indicate that self-identification may function as sufficient, transitional, or concurrent with diagnosis depending on personal meaning-making and structural context. The dominant pattern among self-identified participants was one of self-identification as a stable sufficient endpoint, particularly in the context of systemic barriers and prior clinical invalidation. Among diagnosed participants, the dominant pattern was one in which self-identification preceded and motivated the pursuit of diagnosis, with diagnosis subsequently providing institutional rather than identity-generative functions.

### Co-occurring conditions and identity development

Co-occurring mental health conditions appeared to intersect with identity construction in complex and bidirectional ways. Several participants described long histories of anxiety, depression, PTSD, eating difficulties, ADHD, or sleep-related challenges that had been treated independently before autism was considered. In some cases, these prior diagnoses had obscured autistic traits and delayed recognition, functioning as diagnostic barriers in their own right. As one participant reflected: “Therapy after therapy, and no one ever asked if it could be autism” (Sophia, diagnosed). Another noted: “I’d already been misdiagnosed three times. I stopped trusting the system” (Oliver, self-identified). These accounts illustrate how accumulated misrecognition within clinical systems compounded the difficulty of self-identification and eroded trust in professional assessment.

It is important to clarify the status of these co-occurring conditions as described by participants. In most cases, they referred to actual co-occurring diagnoses documented in Table I, conditions that had been clinically identified prior to any autism assessment. In some instances, however, participants reflected that certain previous diagnoses may have been misattributions of autistic traits: for example, anxiety or ADHD diagnoses that, in retrospect, they understood as manifestations of unrecognised autism. Both situations were present in participants' accounts, and they point to distinct but related processes.

Participants reflected that repeated misdiagnoses reinforced masking behaviours and heightened emotional exhaustion, which in turn exacerbated mental health distress. One participant described this cycle explicitly: “Masking is survival, but it comes at a huge cost” (Zoe, self-identified). Conversely, reinterpreting prior mental health struggles through an autistic framework was described as reducing self-blame and fostering greater coherence in identity narratives. As one participant put it: “Once I stopped seeing myself as defective, everything changed” (Grace, diagnosed). Taken together, these accounts indicate that autism-related identity development and co-occurring mental health trajectories interact bidirectionally: prior diagnoses may delay autistic self-recognition, whilst autistic self-recognition may, in turn, reframe the meaning of prior diagnoses. These processes were particularly pronounced in contexts of delayed or inaccessible formal diagnosis, where participants had relied on personal and community resources rather than clinical pathways to make sense of their experiences.

### Theme 3: masking and emotional exhaustion

All participants reported masking as a survival strategy within neurotypical environments.

*“Every social interaction feels like a performance. I go home exhausted.”* (Jamie, self-identified)

*“Sometimes I don't even know what the 'real me' sounds like anymore.”* (Hannah, diagnosed)

*“I used to rehearse entire conversations before I left the house.”* (Charlotte, self-identified)

Masking was closely linked to burnout, identity confusion, and delayed self-recognition.

*“Letting go of the mask felt terrifying, but freeing.”* (Abigail, self-identified)

*“Masking got me through school, but I lost who I was in the process.”* (Phil, diagnosed)

*“I didn't even realise I was masking until I stopped.”* (Oliver, self-identified)

### Theme 4: barriers to diagnosis and systemic invisibility

Participants across both groups described persistent systemic barriers to recognition, including diagnostic tools designed for children, clinician bias, and long waiting lists.

*“The forms were about toys and tantrums. I'm a 50-year-old woman.”* (Olivia, diagnosed)

*“Autism tests are written for 8-year-old boys, not queer nonbinary adults.”* (Charlotte, self-identified)

*“It took me two years just to get on a waiting list.”* (Grace, diagnosed)

Self-identified participants felt dismissed or excluded by professional systems:

*“Just because I'm articulate doesn't mean I'm not autistic.”* (Lily, self-identified)

*“They said, 'You made eye contact, so you can't be autistic.' I almost laughed.”* (Zoe, self-identified)

*“I was told I was 'too high functioning' for an assessment. What does that even mean?”* (Jamie, self-identified)

### Theme 5: identity development and acceptance

This theme traces the processes through which participants moved from uncertainty to self-acceptance. For some, identity formation followed personal crises or burnout; for others, it emerged gradually through connection and unmasking.

*“It started with a breakdown. But once I found the community, it felt like coming home.”* (Lily, self-identified)

*“I was at rock bottom when I finally put the pieces together.”* (Sophia, diagnosed)

*“It wasn't sudden, it was like peeling back layers.”* (Charlotte, self-identified)

A sense of shared journey and collective validation was central to this process.

*“I found others who said the same things, that sense of not being alone helped me take the next step.”* (Alex, diagnosed)

Despite initial grief over delayed recognition, many described growing pride in their autistic identity:

*“At first I was devastated. Now I see it as one of the best things that's happened to me.”* (Sophia, diagnosed)

*“I'm not hiding anymore. That's freedom.”* (Zoe, self-identified)

*“It took time, but now I wear the label with pride.”* (Alex, diagnosed)

### Theme 6: from pathology to pride: reframing autism

The final theme captures participants' rejection of pathologising narratives and their embrace of neurodiversity-affirming perspectives.

*“I'm not broken, I'm just wired differently.”* (Alex, diagnosed)

*“I don't want to be 'fixed.' I want to be understood.”* (Oliver, self-identified)

*“Autism isn't a tragedy. The way the world treats us is.”* (Sophia, diagnosed)

For many, reframing autism in this way represented an empowering shift from self-doubt to self-acceptance.

*“The word 'disorder' never felt right. This is just who I am.”* (Lily, self-identified)

*“Once I stopped seeing myself as defective, everything changed.”* (Grace, diagnosed)

Together, Themes 1 to 6 trace a coherent trajectory of self-identification, beginning with difference, moving through misrecognition and systemic barriers, and culminating in pride and affirmation.

### ASIS (McDonald, 2017) feedback summary

This subsection summarises participant evaluations of the ASIS (McDonald, 2017), focusing on issues of language, conceptual framing, and inclusivity (See Appendix 1 for further details).

All participants reviewed the ASIS (McDonald, 2017) items, offering detailed feedback on wording, tone, and content. Across both groups, the instrument was perceived as overly deficit-based, outdated, and sometimes stigmatising. Absolute or binary phrasing (e.g., “I have difficulty with…”) was viewed as reductive and misaligned with adult lived experience. Participants consistently preferred identity-first language (e.g., “I am autistic” rather than “I have autism”), arguing that it better captured their sense of self. They also noted the absence of key constructs such as masking, community validation, and the evolving nature of autistic identity:

*“The ASIS doesn't ask about masking. That's huge, it shaped my whole life.”* (Zoe, self-identified)

Based on this feedback, a revised version of the ASIS (McDonald, 2017) was draughted. Eighteen items were reworded to use affirming and inclusive language, and eight new items were added to capture themes such as pride, fluid identity, and social acceptance.

Participants additionally emphasised that the ASIS (McDonald, 2017) could potentially be used as a complementary identity-focused measure to prompt reflective dialogue during adult autism assessments.

While both self-identified and formally diagnosed participants shared the goal of improving inclusivity, subtle differences emerged. Self-identified participants tended to focus on clarity and tone, describing some items as “odd” or “confusing.” They questioned statements implying that autism could be situational or transient (e.g., “I only have autism around certain people”), noting that such phrasing conflicted with their understanding of autism as an enduring aspect of identity. Their suggestions emphasised precision, relatability, and non-pathologizing language.

Formally diagnosed participants engaged more deeply with conceptual framing, objecting to items implying a desire to minimise or cure autism. They viewed such wording as reinforcing social pressure to mask traits. One participant proposed reframing “If I work hard enough, I can minimise my autism” to “If I mask enough, others don't see me as autistic”, highlighting the tension between adaptation and authenticity.

Across both groups, participants underscored the importance of recognising strengths and agency. Both groups agreed that items should acknowledge contextual variation: how environment and societal attitudes shape perceived difficulty rather than autism itself. A strong point of convergence was the rejection of absolute or binary items (e.g., “Autism only makes things harder for me”). Participants considered such statements reductive and inconsistent with a neurodiversity-affirming perspective.

Participants also reported that many commonly used adult assessment tools (originating from child-focused instruments that have later been adapted) often fail to capture how adults experience, interpret, and integrate autism into their sense of self. In this context, they viewed the UK adaptation of the ASIS (McDonald, 2017) as an opportunity to foreground adult voice and contemporary neurodiversity-informed language.

These overlapping yet distinct perspectives emphasise the value of co-production in questionnaire development, ensuring that tools like the ASIS (McDonald, 2017) authentically represent the full spectrum of autistic experience, from self-identification to formal diagnosis.

## Discussion

This study provides insight into the identity formation processes of adults in the UK who either self-identify as autistic or have received a formal diagnosis of autism. Findings from both the focus groups and the ASIS (McDonald, 2017) review illuminate how autistic identity is conceptualised, negotiated, and sometimes constrained by existing assessment frameworks. Together, these datasets emphasise the complexity of self-understanding in autism, the power of language in shaping self-perception, and the value of participatory, affirming approaches in psychological measurement.

### Focus group findings and the social meaning of self-understanding

The focus groups offered rich insight into how neurodivergent adults construct meaning around difference, diagnosis, and belonging. Identity development emerged as a dynamic and relational process shaped by both social context and community validation. Participants oscillated between feelings of difference, acceptance, and ambivalence, echoing identity theories that view selfhood as fluid and negotiated rather than fixed (Allé et al., 2025; Overton et al., 2024).

### Experiencing lifelong difference and masking

For many participants, especially those diagnosed later in life, discovering an autistic identity was transformative yet destabilising (Davies et al., 2024; Dmyterko, 2025). Recognition provided a language to reinterpret lifelong experiences but also prompted grief for years of misrecognition. This ambivalence reflects findings from prior research describing late diagnosis as both liberatory and disorienting (Dmyterko, 2025). Participants also emphasised masking as a central yet exhausting strategy (Edwards et al., 2025). Masking was framed not as individual choice, but as a response to non-accommodating environments, supporting calls to view autistic distress as socially constructed rather than inherently pathological (Garcia-Simon et al., 2025; Scattoni et al., 2021).

Importantly, experiences of masking were frequently intertwined with prior mental health diagnoses and repeated misrecognition within clinical systems. For some participants, prolonged exposure to misdiagnosis contributed to internalised doubt and intensified masking behaviours, reinforcing emotional exhaustion. Conversely, reinterpreting anxiety, depression, or trauma through an autistic framework reduced self-blame and supported greater narrative coherence. These findings suggest that identity development and co-occurring conditions may interact bidirectionally, particularly in contexts of delayed or inaccessible diagnosis.

### Reframing autism through pride and community

Community engagement and peer validation were key catalysts in identity formation. Online spaces and advocacy networks offered both information and belonging, fostering resilience and self-acceptance. These collective processes resonate with social identity theory and neurodiversity scholarship that prioritise interdependence over individualism (Walker, 2021). Taken together, the focus group findings address Research Question 1 by showing that self-identification unfolds as a non-linear, relationally embedded process shaped by three intersecting forces: (1) structural conditions, including diagnostic access, clinician gatekeeping, and service availability; (2) personal meaning-making, particularly through reinterpreting lifelong experiences of difference and masking within an autistic framework; and (3) community belonging, wherein peer validation and shared narrative provided both epistemic resources and identity affirmation. The trajectory was characterised by movement from early, often unnamed experiences of difference, through periods of misrecognition and concealment, towards gradual epistemic self-recognition and, for many, identity integration marked by pride and unmasking (Overton et al., 2024).

The findings also clarify that self-identification encompasses both epistemic self-recognition and identity integration. Participants described first recognising themselves within an autistic framework and later incorporating that understanding into a coherent sense of self. These processes were shaped not only by personal meaning-making but also by structural conditions such as diagnostic access, clinician responses, and social narratives.

### Self-identification in relation to formal diagnosis

Research Question 2 asked how self-identification functions in relation to formal diagnosis, including whether it may serve as a sufficient endpoint or a transitional stage. The findings reveal that both functions were present in the data, with the dominant pattern varying according to diagnostic status. Among self-identified participants, self-identification most commonly functioned as a stable and sufficient identity position, particularly where diagnostic pathways had been experienced as invalidating or inaccessible. Among formally diagnosed participants, self-identification more typically preceded and motivated the pursuit of diagnosis, with diagnosis subsequently providing institutional recognition rather than identity itself. The endpoint/transitional dichotomy therefore has clearest applicability when self-identification precedes diagnosis; where structural barriers prevent diagnosis altogether, self-identification tends to constitute an ongoing, sufficient position rather than a transitional stage per se.

For some self-identified participants, self-identification represented a stable and sufficient identity position, particularly when diagnostic pathways were perceived as inaccessible, invalidating, or unnecessary for self-understanding. For others, self-identification functioned as a catalyst for pursuing formal diagnosis to obtain validation, accommodations, or institutional recognition. Among formally diagnosed participants, self-identification typically preceded diagnosis and facilitated the decision to seek assessment. Diagnosis provided external confirmation and access to support but did not generate identity itself.

These findings support a relational rather than hierarchical model of self-identification and diagnosis. Self-identification and formal diagnosis interact dynamically, and their significance depends on structural context and individual meaning-making. This perspective challenges assumptions that self-identification is inherently provisional or that diagnosis is inherently definitive.

### Interpretation of ASIS (McDonald, 2017) feedback

Participant feedback on the ASIS (McDonald, 2017) revealed consistent concerns about its deficit-oriented and medicalised language. Both self-identified and formally diagnosed participants rejected phrasing implying that autism should be “minimised” or “cured,” highlighting the dissonance between clinical discourse and lived experience. This finding extends previous work suggesting that deficit-based terminology reinforces internalised stigma and limits identity development (Lilley et al., 2022; Lopez et al., 2022).

Differences in emphasis between groups address Research Question 2 further, showing how diagnostic status shapes engagement with identity measures. Self-identified participants sought clarity and resonance, reflecting the tentative, exploratory nature of self-definition (Overton et al., 2024). Formally diagnosed participants, meanwhile, critiqued conceptual assumptions more directly, advocating for affirming, identity-first language and rejecting deficit-based framing (Scattoni et al., 2021).

Participants from both groups rejected absolutist items such as “Autism only makes things harder for me,” viewing them as reductive and incompatible with a strengths-based perspective. The revised ASIS, adding items on masking, pride, and community validation, thus represents not merely linguistic refinement but a conceptual shift toward participatory, identity-based measurement (Pukki et al., 2022; Sonuga-Barke et al., 2024). This addresses Research Question 3 by illustrating how collaborative critique can inform more inclusive psychometric design.

Importantly, participants did not advocate replacing established diagnostic instruments. Rather, they positioned the ASIS (McDonald, 2017) as potentially useful as a complementary identity-focused measure that could prompt reflective dialogue within adult assessment contexts. This distinction reinforces the non-diagnostic nature of the ASIS and situates the UK adaptation as a contribution to person-centred assessment rather than a substitute for NICE-recommended tools. The co-production process itself constitutes a meaningful demonstration of the value of designing psychological instruments in dialogue with the communities they aim to represent, not merely as an abstract principle, but as a practice that yielded concrete, substantive revisions to the scale.

### Integration of datasets and theoretical implications

When considered together, the focus group data and ASIS (McDonald, 2017) feedback provide a multilayered account of autistic self-understanding. The former shows internal and social processes of meaning-making; the latter reveals how individuals engage critically with external representations of identity. Their convergence underscores that self-definition among autistic adults is dialogically constructed through interaction between personal experience and institutional discourse (Pellicano & Den Houting, 2022; Petty et al., 2023; Pritchard-Rowe et al., 2025).

Measurement tools such as the ASIS (McDonald, 2017) are not neutral artefacts; they embed epistemic assumptions that participants actively contest. Co-revision of such instruments thus becomes an act of epistemic justice, repositioning autistic adults as contributors to psychological knowledge rather than passive recipients of evaluation (Lerner et al., 2023; Maitland et al., 2021; Russell et al., 2025).

### Methodological reflections

Integrating autistic perspectives into the revision of the ASIS (McDonald, 2017) highlighted both the promise and challenges of participatory methodology. Engaging participants as co-authors required flexibility and reflexivity, particularly when feedback appeared inconsistent with traditional psychometric norms. Divergent or critical perspectives revealed conceptual depth, reflecting the diversity of autistic worldviews. Narrative exploration within focus groups likewise captured the fluid and sometimes contradictory nature of autistic identity, demonstrating the limits of standardised measurement (Krueger & Casey, 2015; Reisner et al., 2018).

Participatory research, however, is not immune to power imbalances. Although this study employed iterative feedback, transparent coding, and collaboration with autistic co-researchers, tokenism remains a risk. Ongoing reflexivity is essential to ensure that participation translates into genuine influence rather than symbolic inclusion.

Overall, the findings converge to show that self-identification is not a static endpoint but a dynamic, socially embedded process that interacts with diagnostic systems, community discourse, and the language of measurement itself (Allé et al., 2025; Cooper et al., 2021; Davies et al., 2024). By combining thematic and item-level analyses, this study advances understanding of autistic identity development and demonstrates the importance of co-produced, neurodiversity-affirming approaches to psychological assessment (Sonuga-Barke et al., 2024).

## Limitations

Despite its contributions, the study presents several limitations. The participant sample was relatively small, verbally able, and demographically limited, with most identifying as White, educated, and female. Because participation involved focus groups and online discussion, the study likely favoured individuals with higher verbal fluency, digital literacy, and social confidence. This composition constrains the generalisability of the findings but simultaneously offers valuable insight into a group often marginalised or misrecognised within autism research.

The over-representation of women and gender-diverse participants likely reflects broader diagnostic biases: these groups are among the least likely to receive timely or accurate identification, making self-recognition a particularly salient route to understanding autism. Furthermore, self-selection bias may have favoured individuals already engaged with neurodiversity discourse, potentially amplifying affirming perspectives.

We recognise that some groups are likely underrepresented here, including adults with severe or complex co-occurring conditions, people with significant intellectual disability, non-verbal autistic adults, older adults, people from minoritised ethnic backgrounds, and those with limited digital access. Future research should purposively recruit such groups and consider tailored methods.

Additionally, the separation of participants by diagnostic status, while analytically useful, may have simplified the fluidity with which some individuals move between self-identification and formal diagnosis over time. Longitudinal designs would allow more nuanced examination of how these processes evolve and interact.

Finally, while the revised ASIS (McDonald, 2017) represents a significant linguistic and conceptual improvement, it requires empirical validation with larger and more diverse populations to evaluate its psychometric robustness. Full psychometric validation of the UK version (including factor analysis, reliability and validity testing) will be conducted and reported in a subsequent publication before clinical or research application.

Moreover, while participants articulated how identity-focused measures might complement clinical assessment, this study did not evaluate the practical integration of the ASIS (McDonald, 2017) within diagnostic pathways. Future research could explore how such tools function in real-world assessment settings and whether they meaningfully enhance person-centred dialogue.

## Implications and future directions

While exploratory and based on a modest sample, this study contributes meaningfully to the growing body of work on neurodiversity-affirming assessment. By incorporating autistic voices into the UK adaptation and interpretation of the ASIS (McDonald, 2017), it models a participatory approach that challenges traditional hierarchies of expertise.

Specifically, participant feedback highlights three areas for potential refinement of self-identification tools: (1) simplifying and de-clinicalising item wording to enhance everyday relevance and comprehension; (2) broadening item content to capture phenotypic diversity (including female-typical presentations, later recognitions, and variation linked to cognitive profile); and (3) clarifying response formats to reduce ambiguity. These insights are grounded in qualitative feedback (see Appendix 1), but require empirical testing.

Importantly, the findings also suggest that identity-focused measures may serve a distinct function from behavioural diagnostic instruments. Rather than determining eligibility, such tools may facilitate reflective exploration of strengths, challenges, and contextual influences. Recognising this distinction may help clinicians integrate identity-oriented dialogue into assessment processes without conflating identity measurement with diagnostic determination.

Beyond the UK version of the ASIS (McDonald, 2017), these findings have broader implications for clinicians and educators. Professionals using self-assessment tools should recognise how phrasing and framing influence respondents' sense of self. Training programmes in psychology and allied disciplines could embed participatory design principles, encouraging practitioners to view autistic individuals not merely as subjects of study but as co-producers of knowledge.

## Conclusion

This study contributes to understanding how neurodivergent adults construct and negotiate identity within social, diagnostic, and linguistic contexts. Through participatory engagement with the ASIS (McDonald, 2017), it demonstrates that measurement tools shape as well as describe experience, influencing how identity is recognised and validated.

Rather than offering a definitive model of autistic identity, the study underscores the value of designing psychological instruments in genuine dialogue with the communities they aim to represent—a process that in this case yielded substantive improvements to the ASIS and affirmed the importance of identity-affirming language in clinical and research contexts. Recognising autistic identity as dynamic, relational, and co-constructed invites a shift in both research and practice, from assessing autism as difference to understanding it as an evolving expression of human diversity.

By conceptualising self-identification as encompassing both epistemic self-recognition and identity integration, and by situating it in relation to formal diagnosis rather than in opposition to it, this study contributes a relational framework for understanding autistic self-definition in adulthood.

## Data Availability

Anonymized summary data that support the findings of this study are available from the corresponding author upon reasonable request.

## Appendixes

### Appendix 1. Participant feedback on the ASIS (McDonald, 2017)

**Table:**

Initial items	Self-identified participants		Participants with a formal diagnosis of autism		Final items
	Comments	Suggestions	Comments	Suggestions	
1. I feel like I only have autism in certain activities, like completing work, organising, getting ready to go somewhere, or new activities.	From my awareness autism relates to every aspect of my life, highlighting areas I am really strong, proficient and excel in consistently. It is a very vague question to ask that confuses me. Yes, this is true for me, there are activities (such as programming)	Prefer to remove the word ‘feel’. Prefer to see the statement read, for example, what particular areas of your life do you struggle with? E.g., making new friends, keeping friends, socialising, having a boyfriend, girlfriend, partner, cooking and eating healthy and nutritious food, washing, bathing and keeping myself clean etc		No suggestions given by participants	1. I experience autism in various parts of my life, including tasks like organising, socialising, or trying new things.
2. There is little I can do about autism	This to me seems a very patronising and defeated statement, as if I am defective in some way, very derogatory where there seems to be a connation of shame attached to it. Unfortunately, I think my brain fog is impending my ability to compensate for some of my communication deficits, so there are things that could be done, but I am not able to put them into practice (as it mostly involves reasoning about things).	No suggestions given by participants		No suggestions given by participants	2. I sometimes struggle with certain aspects of autism, especially with things like communication, but I am always learning and adapting.
3. I am good at some things because I have autism.	The statement needs to remove the word ‘because’. I would not use autism as an excuse or justification with the word ‘because’ I think it makes me better in some respects in helping others as I tend to be thoughtful and logical rather than responding emotionally (which seems to be a good way of initiating action, but not of selecting a productive action).	Prefer to see the statement reworded as a question to invite more of a dialogue of specific gifts. For example, “what hobbies, interests, skills and talents are you great at and you find easy and fun??	The statement is a repetition of statement 15. The statement resonates with me-	No suggestions given by participants.	3. I am skilled at certain things that I enjoy, such as being logical and thoughtful in helping others.
4. There are some people with whom I don’t feel I have	Yes, I would say so.	No suggestions given by participants		No suggestions given by participants	4. There are certain people or environments where my autism feels less pronounced.
5. Autism only makes things harder for me	No, I would say that I am exceptionally gifted at many things including being clear, direct and blunt in communication. This is what I find hard, trying to bend and contort myself to please and appease others. No, I find absolute statements like this rather difficult as it probably isn’t meant to mean that absolutely nothing is better in anyway even to a minimal degree, so I need to gauge what the intended meaning actually was.	No suggestions given by participants.		No suggestion given by participants.	5. I face challenges because of autism, but I also have developed ways to work with those challenges.
6. I like having autism or being autistic	The statement resonates with me	No suggestions given by participants	The statement resonates with me. The statement is a repetition of statement 12.	No suggestions given by participants	6. I embrace my autism as an important part of who I am.
7. My good qualities have little to do with autism	The statement is vague. What is meant by “qualities?” Some could be due to autism and others from nature/nurture.	No suggestions given by participants.	The statement is a repetition of statements 19 and 22. The statements mean the same thing.	No suggestions given by participants.	7. Some of my strengths may be related to autism, while other come from different life experiences or personal traits.
8. I feel like I only have autism around certain people, like classmates, teachers, parents, or co-workers.	My communication difficulties stem around people who show little or no respect. I am more aware of it (being autistic) with some people (mostly my wife), but it is apparent to some extent with most people	No suggestions given by participants.	Some of the statements feel like “autism” is a blanket ailment and a negative thing to have.	Prefer to see the statement read “I only feel I have difficulties around certain people, e.g., classmates/parents/teachers/co- workers.”	8. I tend to experience more difficulties related to being autistic when interacting with certain people (e.g., classmates, teachers, parents, or co-workers)
9. I feel autism has more benefits in abilities than challenges	I perceive the challenges come from trying to fit into a medical model system, rather than society adapting to an individualised social model system. More benefits will arise when education is embedded more in all sectors of life, and stigma, judgement, separations, expectations and prejudices are irradicated from society. No.	No suggestions given by participants.		No suggestions given by participants.	9. I believe autism brings many strengths, and that most challenges come from how society responds to difference.
10. If I work hard enough, I can minimise my autism.	The statement almost encourages masking. Masking has Detrimental repercussions for health (e.g., breakdown and burnout). I find the concept of “minimising” my autism highly insulting, like it is something to be ashamed and embarrassed about. The statement is a difficult one, as it seems a tautology.	No suggestions given by participants.	The statement is about masking. I don’t think there is such a thing as “minimising” autism– only how it’s perceived in response to making ability, whilst hiding the detrimental health effects underneath.	Prefer to see the statement read “If I mask enough, others don’t see me as autistic.”	10. People often don't see me as autistic because I've learned to mask certain traits
11. I would be better off if I didn’t have autism.	Better off financially? emotionally? mentally? spiritually? all or some connected? I find this a shamefully embarrassing statement, that perceives a sense of guilt for not being perfect. I am choosing to make no apology for me being me anymore and I love my traits of difference. Yes	No suggestions given by participants.		No suggestions given by participants.	11. Sometimes I wonder if life would be easier without the challenges that come with being autistic,
12. I like the way I am different from everyone else.	1,000,000% true for me! No.	No suggestions given by participants	The statement is a repetition of statement 6. I would say the statements mean the same thing.	No suggestions given by participants	12. I feel proud of the ways I experience and interact with the world differently from others.
13. I feel like I only have autism in certain places, like school, home, work or somewhere new	I am selectively around certain people and places of interaction (I am aware of hidden agendas).	Some places are worse than others, especially ‘somewhere new’.	The statement feels like ‘autism’ is a blanket ailment. The statement feels like autism is a negative thing.	Prefer to see the statement read “I only feel I have difficulties in certain environments.”	13. New or unfamiliar environments often make my autistic traits more noticeable or challenging.
14. When I’m alone, I don’t feel like I have autism.	The statement is really odd and does not make sense. I am not sure how autism is supposed to feel! I’m just reminded of it (being autistic) less when I’m alone.	No suggestions given by participants	The statement feels like ‘autism’ is a blanket ailment. The statement feels like autism is a negative thing. The statement resonates with me	Prefer to see the statement read “When I’m alone, I feel better because there are no sensory or social demands.”	14. Being alone helps me feel more at ease because I'm not managing social or sensory challenges.
15. Autism means having unique abilities.	1,000, 1,000% true for me—superpower gifts of magnitude that we are rarely acknowledged in this reality and society, other than perceived as ‘odd’, ‘crazy’, ‘weird’ to those who judge. I don’t think that this is the case (apart from an ability to have focused interests will inevitably make you better at those interests than you would otherwise be, possibly to the detriment of other things).	No suggestions given by participants.	The statement is a repetition of statement 3. The statement resonates with me.	No suggestions given by participants.	15. For me, being autistic includes having unique abilities that shape how I think and interact with the world.
16. If I work hard enough, I can minimise the challenges associated with autism.	The statement is a repetition of statement 10. The statement is very much like statement 10, see response to that.	No suggestions given by participants.	The statement resonates with me.	No suggestions given by participants.	16. How challenging autism feels often depends on the environment I'm in and the support I receive.
17. There are some places where I don’t have Asperger’s/autism	It sounds like an ambiguous statement.	I would remove the word ‘feel’.	The statement feels like “autism” is a blanket ailment, which could cover a multitude of characteristics and portrayed as a negative thing to have (and there are so many variations of how people experience it).	Prefer to see the statement read ‘find it easier’.	17. There are places where I don’t experience as many challenges related to being autistic
18. If I were cured of autism, I wouldn’t be me anymore.	The statement sounds like autism is a disease, which it is not. The statement comes across as highly discriminatory. The statement is always logically true, so the intended meaning is not clear.	The statement requires removing completely because it is discriminatory. Prefer to see the statement read “Is autism an important aspect of my identity?”	The statement feels like it’s promoting a damaging stereotype giving the notion that autism could be cured.	Prefer to see the statement read “If I wasn’t autistic, I wouldn’t be me.”	18. Being autistic shapes how I think, feel and experience the world, it's a core part of me.
19. I don’t feel I have additional abilities from my autism.	I have no idea what this statement means. The statement resonated with one participant.	Perhaps use the word ‘think’ or ‘consider’ instead of ‘feel’. Prefer to see statement read “What other additional abilities would you say you have?”	The statement is a repetition of statements 7 and 19. The statements mean the same thing.	No suggestions given by participants.	19. I believe being autistic gives me strengths that others may not have.
20. I only “have autism” when people treat me like I do.	The statement is really odd. I have no answer for the statement. No.	No suggestions given by participants.	The statement feels like ‘autism’ is a blanket ailment. The statement feels like autism is a negative thing.	Prefer to see statement read “I only feel autistic when people make light of my difficulties/differences”.	20. Other people's reactions sometimes make me more aware of being autistic
21. I am better off because I have autism.	The statement is a repetition of statement 11. What is meant by better off? better off financially? emotionally? mentally? physically? spiritually? all? or some connected?	No suggestions given by participants.		No suggestions given by participants.	21. In some ways, I am better off because I have autism (e.g., emotionally, intellectually, socially)
22. My strengths have little to do with autism.	The statement is ambiguous. The statement is a repetition of statement 7.	No suggestions given by participants.	The statement is a repetition of statements 7 and 19. The statements mean the same thing.	No suggestions given by participants	22. Some of my strengths are unique to my experience as an autistic person
Extra item suggested by participants: I have not felt the need to hide or minimise my autism.					23. I have not felt the need to hide or minimise my autism.
Extra item suggested by participants: Being autistic is sometimes not recognised or adequately assessed by healthcare professionals or diagnostic tools.					24. Being autistic is sometimes not recognised or adequately assessed by healthcare professionals or diagnostic tools.
Extra item suggested by participants: I've noticed that I experience the world differently from others					25. I've noticed that I experience the world differently from others
Extra item suggested by participants: Along with autism, I have been diagnosed with other psychological conditions					26. Along with autism, I have been diagnosed with other psychological conditions.
Extra item suggested by participants: I have other physical health conditions in addition to autism.					27. I have other physical health conditions in addition to autism.
Extra item suggested by participants: I sometimes tell people that I think I might be autistic.					28. I sometimes tell people that I think I might be autistic.
Extra item suggested by participants: As an adult, being autistic is often overlooked in society, where there is still limited awareness and understanding.					29. As an adult, being autistic is often overlooked in society, where there is still limited awareness and understanding.
Extra item suggested by participants: Being autistic may not be immediately recognised due to demographic factors, such as gender or age, which can influence how autism is perceived. .					30. Being autistic may not be immediately recognised due to demographic factors, such as gender or age, which can influence how autism is perceived.
General comments	Autism is perceived differently to different people also and I cannot always articulate in words what I want to say. More specific and direct questions would help to be clearer.		I’m not sure if the scale covers the reasons or thoughts that made me think I might be autistic.		
			The only thing I knew was that something was definitely wrong.		
			For me, it wasn’t just one thing—it was a buildup of years of moderate mental health struggles, physical tics, social anxiety, and behaviours that didn’t fit.		
			I was achieving a lot, but feeling like I was getting nowhere. I’d get overwhelmed by environments and sensory input, which led to trouble keeping jobs or being involved in social situations. The stress would build up quickly, and I’d burn out.		
			For me, it was always more about my way of thinking—this didn’t feel like just something physical.		
			When I first looked at this in 2016, I felt like the language and terminology used were really outdated. I think we’ve made a lot of progress in understanding autism and the spectrum since then.		
			While I get that the statements need to be simple and direct, I couldn’t help but notice how autism is described as something you “have.” It feels a little off, almost like it’s something temporary you can get rid of, like a cold or a bad tooth! The only exception was statement 6, which was worded both ways—so it didn’t really match the rest of the statements.		
			From what I see in forums and social media, many people on the spectrum prefer “identity-first” language, where we say we are “autistic” rather than saying autism is something we “have.” It feels more accurate to me because autism is part of who I am—it’s not something that can be cured or taken away.		
			Of course, self-identity is personal, and some might prefer the “having” language. But to me, that seems outdated and makes it sound like autism is a separate condition—almost like it’s something that can be treated or removed.		
			I also noticed some repetition in the statements—like 3 & 15, 6 & 12, and 7 & 19, 22, which pretty much say the same thing. On top of that, the whole scale feels a little confusing.		
			A lot of the statements seem to overlap in meaning but are worded differently. Plus, they jump from positive to negative affirmations. If the order was adjusted to have a better flow, it might make it easier to follow.		

### Appendix 2. Thematic analysis summary: themes, subthemes, and representative quotations

**Table:**

Theme	Subtheme/Code	Representative quotations (Participant)
1. Experiencing Difference in a Neurotypical World	Lifelong sense of difference	“*I always felt like I missed a memo that everyone else got about how to be a person*.” (Zoe, self-identified) “*Even as a kid, I thought something was ‘off,’ but no one else saw it*.” (Phil, diagnosed) “*It was like everyone had the rulebook but me*.” (Charlotte, self-identified)
	Feeling out of sync/alienation	“*I’ve always felt like an alien trying to learn the rules of a planet I wasn’t from.”* (Alex, diagnosed) “*I learned to mimic people just to pass as normal.”* (Lily, self-identified) “*I was always watching, never really joining in*.” (Jamie, self-identified)
	Early social confusion	“*I didn’t get why friendships worked for everyone else but not me.*” (Olivia, diagnosed) “*I was punished for things I didn’t even know were wrong.”* (Sophia, diagnosed)
2. Diagnosis and the Construction of Identity	Validation through diagnosis	“*Diagnosis gave me a language for what I’d always felt but couldn’t explain.*” (Grace, diagnosed) “*It validated everything I’d been gaslit into doubting.”* (Phil, diagnosed)
	Autonomy and self-knowledge	“*I don’t need a certificate to know who I am.*” (Charlotte, self-identified) “*I’d already done all the research before the doctor confirmed it.*” (Zoe, self-identified)
	Misdiagnosis and distrust	“*Therapy after therapy, and no one ever asked if it could be autism*.” (Sophia, diagnosed) “*My GP literally said, ‘You don’t look autistic.’ That killed it for me.*” (Lily, self-identified) “*They told me I was too social to be autistic. Whatever that means.*” (Jamie, self-identified)
	Gatekeeping and exclusion	“*I’d already been misdiagnosed three times. I stopped trusting the system*.” (Oliver, self-identified) “*I was told I was too high-functioning for an assessment.*” (Jamie, self-identified)
3. Masking and Emotional Exhaustion	Concealment and performance	“*Every social interaction feels like a performance. I go home exhausted*.” (Jamie, self-identified) “*Sometimes I don’t even know what the ‘real me’ sounds like anymore*.” (Hannah, diagnosed)
	Identity confusion and loss	“*Masking got me through school, but I lost who I was in the process*.” (Phil, diagnosed) “*I became so used to performing that I forgot who I actually was*.” (Olivia, diagnosed)
	Liberation from masking	“*Letting go of the mask felt terrifying, but freeing*.” (Abigail, self-identified) “*I only felt like myself once I stopped pretending*.” (Charlotte, self-identified)
	Emotional cost and burnout	“*Masking is survival, but it comes at a huge cost*.” (Zoe, self-identified) “*I can’t keep performing all the time; it breaks you down.”* (Oliver, self-identified)
4. Barriers to Diagnosis and Systemic Invisibility	Inaccessible diagnostic tools	“*The forms were about toys and tantrums. I’m a 40-year-old woman*.” (Olivia, diagnosed) “*It felt like the assessment was written for children, not adults*.” (Sophia, diagnosed)
	Clinician bias	“*Autism tests are written for 8-year-old boys, not queer nonbinary adults*.” (Charlotte, self-identified) “*They looked surprised because I made eye contact*.” (Grace, diagnosed)
	Service delays and exclusion	“*It took me two years just to get on a waiting list*.” (Grace, diagnosed) “*By the time I got the assessment, I’d already self-diagnosed*.” (Lily, self-identified)
	Invalidating professional responses	“*Just because I’m articulate doesn’t mean I’m not autistic*.” (Lily, self-identified) “*They said I was too empathetic to be autistic*.” (Abigail, self-identified)
5. Identity Development and Acceptance	Catalysts to self-awareness	“*TikTok was the lightbulb moment. Suddenly everything made sense*.” (Lily, self-identified) “*Reading about female autism profiles was like reading my diary*.” (Olivia, diagnosed)
	Peer validation and belonging	“*I found others who said the same things; that sense of not being alone helped me take the next step*.” (Alex, diagnosed) “*Finding community gave me words I never had before*.” (Charlotte, self-identified)
	Emotional reconciliation	“*It started with a breakdown, but once I found the community, it felt like coming home*.” (Lily, self-identified) “*I was angry at first—why did no one see it sooner?*” (Sophia, diagnosed)
	Emergence of pride	“*At first I was devastated. Now I see it as one of the best things that’s happened to me*.” (Sophia, diagnosed) “*It took time, but now I wear the label with pride*.” (Alex, diagnosed) “*I’m not hiding anymore. That’s freedom*.” (Zoe, self-identified)
6. From Pathology to Pride: Reframing Autism	Rejecting deficit narratives	“*I’m not broken, I’m just wired differently*.” (Alex, diagnosed) “*I don’t want to be ‘fixed.’ I want to be understood*.” (Oliver, self-identified)
	Reclaiming language	“*The word ‘disorder’ never felt right. This is just who I am*.” (Lily, self-identified) “*Autism isn’t a tragedy. The way the world treats us is*.” (Sophia, diagnosed)
	Integration and growth	“*At first I thought of autism as something wrong with me; now I see it as something that makes sense of who I am*.” (Alex, diagnosed) “*Once I stopped seeing myself as defective, everything changed*.” (Grace, diagnosed)
	Pride and advocacy	“*I used to hide my diagnosis; now I correct people who talk about curing autism*.” (Hannah, diagnosed) “*Being autistic is just another way of being human*.” (Jamie, self-identified)
